# Knowledge-Based Dietary Intake Recommendations of Nutrients for Pediatric Patients with Maple Syrup Urine Disease

**DOI:** 10.3390/healthcare11030301

**Published:** 2023-01-18

**Authors:** Mayda Alrige, Haneen Banjar, Taghreed Shuaib, Amal Ahmed, Raghad Gharbawi

**Affiliations:** 1 Information Systems Department, Faculty of Computing and Information Technology, King Abdulaziz University, Jeddah 21577, Saudi Arabia; 2 Computer Science Department, Faculty of Computing and Information Technology, King Abdulaziz University, Jeddah 21577, Saudi Arabia; 3 Center of Artificial Intelligence in Precision Medicine, King Abdulaziz University, Jeddah 21577, Saudi Arabia; 4 Department of Genitics Medicine, Faculty of Medicine, King Abdulaziz University, Jeddah 21577, Saudi Arabia

**Keywords:** artificial intelligence, knowledge base, maple syrup urine disease, recommended dietary intake, pediatric patients

## Abstract

Maple syrup urine disease (MSUD) is a metabolic disorder characterized by a difficulty to digest and process proteins necessary for growth. To monitor and maintain the ideal growth of children with MSUD, caregivers need to carefully control the consumption of harmful branched-chain amino acids (BCAAs). The dietary limits of amino acids for MSUD patients are recommended and controlled by pediatricians and metabolic dietitians according to age, height, weight, and the prevailing percentage of amino acids in the body. This study introduces an intelligent dietary tool called MSUD Baby Buddy for caregivers of MSUD patients that tracks the amino acids intake out of baby formulas for babies 0–6 months old. This tool aims to provide accurate recommendations of the appropriate daily intake of protein and BCAAs based on the patients’ data, plasma BCAAs, and formula preferences. We use a knowledge-based system, including knowledge acquisition and verification, as well as knowledge management tool validation, and the ripple-down rules are employed for building the system. MSUD Baby Buddy can support the maintenance of adequate amino acid levels and increase awareness about the control of BCAAs. The average usability of MSUD Baby Buddy is 84.25, indicating that the tool is intuitive and may help caregivers to easily determine the recommended doses of formula based on patients’ biometric data and preferred formula. On the other hand, interviews with metabolic dietitians revealed some drawbacks, which were addressed to further improve the tool. MSUD Baby Buddy is expected to help caregivers of MSUD patients to independently track nutrient intake and reduce the number of visits to the pediatrician and metabolic dietitian.

## 1. Introduction

Maple syrup urine disease (MSUD) is a rare inborn error of metabolism (IEM), with an estimated 1 in 185,000 newborns being diagnosed with this disease worldwide [1]. A patient with MSUD cannot correctly break down proteins that are essential for growth, development, and tissue repair. For a body to gain nutrition from consumed food, they must be broken down into the constituent amino acids. However, MSUD patients cannot break down the three branched-chain amino acids (BCAAs), namely, valine, leucine, and isoleucine, from consumed food. This inability causes harmful substances to accumulate in the blood and urine, leading to metabolic crises and learning difficulties. If MSUD is left untreated, patients will likely deteriorate, slip into a coma, and possibly die. Therefore, caregivers and specialists must care for and monitor the health of MSUD patients, especially aiming to control and reduce dangerous levels of BCAAs within the patients’ bodies [2].

MSUD patients must follow a strict low-protein diet to control and reduce the intake of BCAAs while maintaining adequate levels of protein, fluid, and energy for proper growth and development. Tracking the daily intake of BCAAs is challenging because their contents are not readily available on food labels or nutrition tables. Parents and guardians normally consult pediatricians or metabolic dietitians to obtain recommendations for nutrient content quantities [3]. The BCAA levels that can be tolerated by children depend on age, height, weight, and BCAA concentration in blood plasma. As babies grow rapidly in their first 6 months, the recommended quantities must be reassessed every 2–4 weeks, and dietary nutrient content must be adjusted accordingly. Furthermore, parents or caregivers are always at risk of under-/overestimating the amino acid content of formula, threatening metabolic control [4]. To mitigate this issue, diet tracking has been studied for many diet-related chronic diseases [5], not only inherited metabolic chronic diseases [6]. Mobile health and dietary management systems are available for diet tracking to guide individuals based on certain dietary recommendations and nutrition protocols [5]. For instance, the Metabolic Diet App is an online web application for patients with IEM, covering 15 types of IEMs. The personalized dashboard and management plan for tracking and planning meals include an input for homemade recipes, a food diary for managing and tracking daily intake, and a tracker of BCAA intake [6]. A similar mobile application (app) is Diet Assistant, which is a leucine calculator for MSUD patients, in which users can track their daily intake of leucine by registering every meal they eat on the app registry. These inputs are exported to a daily log for users to check. In addition, the app has a progress bar that indicates the amount of leucine consumed within the daily limit [7]. All these apps are great attempts to help to track diet and recommend nutrient intake for different chronic metabolic disorders; however, they fall short of translating the certain nutrient quantities into practical, meal-based daily limits, as caregivers often struggle to transfer the daily limit into formula doses throughout the day. Many parents and guardians fail to evaluate the amino acid limits according to the patient’s age, weight, and plasma BCAAs, and consequently cannot apply these limits to the baby formula daily dosages.

To address this problem, we introduce MSUD Baby Buddy, an intelligent dietary tool, with the main aim of providing accurate recommendations of the appropriate daily intake of protein and BCAAs in the right amount of formula daily dosages. These recommendations take into account patients’ data, plasma BCAAs, and formula preferences. The proposed knowledge-based system can be used for tracking diets, helping caregivers of MSUD patients 0–6 months old to personalize diets based on biometric data. MSUD Baby Buddy provides easy formula dosage recommendations for MSUD patients based on weight, age, and plasma BCAAs while considering medical and commercial formula preferences. The general recommended nutrient content is based on gold-standard nutrition support protocols used by pediatricians and metabolic dietitians [8]. In addition, MSUD Baby Buddy provides customized nutrition recommendations based on formula preferences. MSUD Baby Buddy is intended to achieve the following objectives:

1. Calculate the recommended daily intake of fluid, protein, energy, and BCAAs (i.e., leucine, isoleucine, and valine) based on the patient’s BCAA levels, age, and weight;
2. With a rule-based system, recommend doses of medical and commercial formulas depending on BCAAs, fluid, protein, and energy using the calculated daily intake limits;
3. Provide general health tips for caregivers to maintain the metabolic health of MSUD patients.

## 2. Materials and Methods

Software-based research has been conducted to develop and evaluate the knowledge-based MSUD Baby Buddy app, which was developed as a handy app that can be easily installed on the mobile phones of caregivers. This app assists in developing a knowledge base of the MSUD patient and includes steps of knowledge acquisition, verification, and knowledge management tool validation. The ripple-down rules (RDRs) are used for building the system to support the diet of MSUD patients [9]. The app considers five variables: age, height, weight, plasma BCAAs, and type of formula (both commercial and medical). The variables and desired quantity of daily protein are used to recommend adequate BCAA intake to caregivers. For example, when the caregiver provides the patient’s preferred formula and data, MSUD Baby Buddy presents the recommended daily food intake (formula dosage).

### 2.1. The Knowledge-Based System

**Figure 1:**
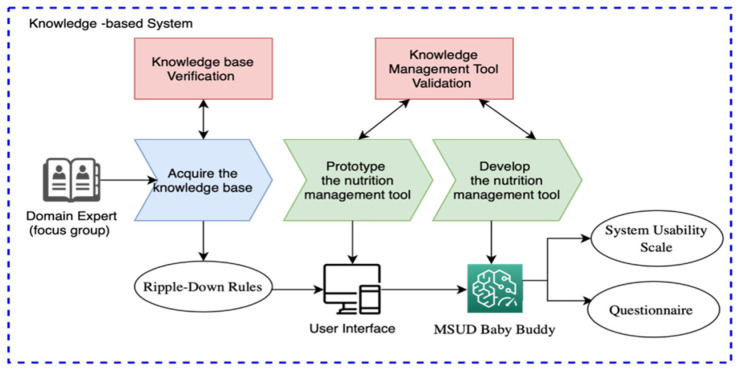
Flowchart to develop and evaluate knowledge base of MSUD Baby Buddy.

The flowchart to develop and evaluate the knowledge base of MSUD Baby Buddy is shown in Figure 1. The first phase concerns knowledge acquisition, where the knowledge base has been built using the RDRs, which is elaborated on in Section 3.2. The second phase is for knowledge management and validation, where, first, the nutrition management tool has been prototyped, elaborated on in Section 3.3; and, second, the full version of the tool (MSUD Baby Buddy) has been developed, elaborated on in Section 3. An open-ended questionnaire was used to test and evaluate the tool by parents, guardians, caregivers, and nursing or health care professionals of MSUD patients. Ethics approval was obtained from the Unit of Biomedical Ethics of the King Abdulaziz University Hospital (No. 229-21).

### 2.2. Knowledge Acquisition

**Figure 2:**
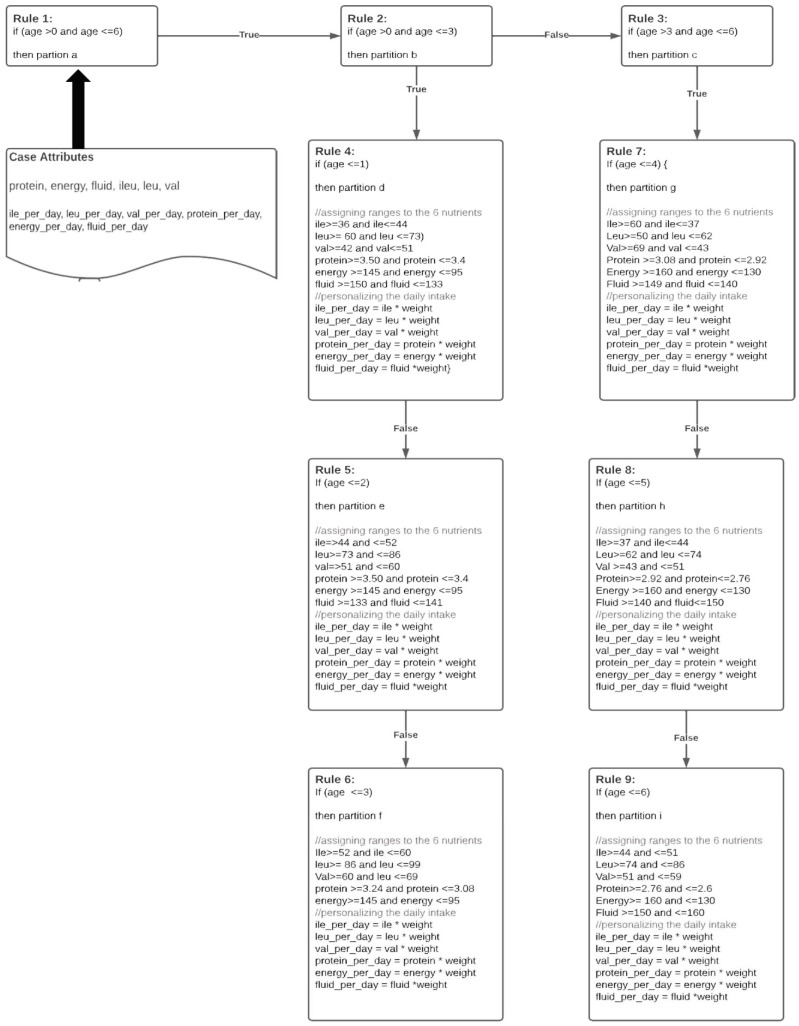
Knowledge acquired using RDRs [10].

Diet tracking has been widely studied, and diverse knowledge-based systems are available in medicine to recommend solutions for individuals based on certain protocols. The proposed MSUD Baby Buddy follows the RDRs to acquire and build the knowledge base [10]. Knowledge acquisition was aimed to create and refine rules, as depicted in Figure 2. An RDR allows a domain expert to define the processing of incoming information for decision making. In addition, RDRs incrementally subdivide the object space into smaller partitions, aiming to classify all the objects from the same class to the same partition. This partitioning can be established by the domain expert, ensuring an adequate subdivision [9].

### 2.3. Knowledge Verification

Then, we verified the knowledge acquired in the first step. This process took approximately 5 months, from 20 August 2021 to 21 January 2022. The MSUD nutritional facts were used to identify initial allowable limits of BCAAs and protein intake based on the patient’s age and weight [9]. A metabolic dietitian assisted in acquiring the details of the variables to provide a recommendation. The age variable was categorized into six different groups to meet the BCAA requirements, being classified on a monthly basis from 0 (birth) to 6 (half a year) months. Finally, we conducted a focus group discussion, with the participation of three metabolic dietitians and nutritionists/specialists, about formulating rules regarding types of formulas and units before recommending the type and quantity of formula for every patient.

### 2.4. Knowledge Management and Validation

After verifying the knowledge base, we implemented user interfaces for the MSUD Baby Buddy app. We aimed to validate and verify the usability and adaptability of the created interfaces. Usability testing allows measurement of the ease of handling and clarity in understanding an app. As these attributes vary according to the user and personal knowledge, the attitude of the participants was noted to understand the easiness, simplicity, and friendliness of the interfaces and app [10].

We adopted the system usability scale (SUS) for testing usability [11]. The SUS is reliable in the practical application and evaluation of various kinds of application systems. As the MSUD Baby Buddy app is intended for use worldwide, it must meet usability parameters in line with MSUD nutrition protocols. The same standard and ease of use is required for adaptability and performance of required tasks by caregivers.

After polishing and confirming the interfaces and reaching consensus about MSUD Baby Buddy usability, we started working on the back end of the app. Finally, we deployed unit testing according to relevant ethical procedures to evaluate the main functionalities of MSUD Baby Buddy [12].

## 3. Results

This section presents the results of the knowledge acquired using RDR. The second subsection presents the results of verifying with domain experts the dietary recommendations and nutrition protocols. The third subsection presents the results of tool validation through front-end and back-end testing.

### 3.1. Acquired Knowledge 

By obtaining the partitioning scheme and rules from the domain experts, we represent the knowledge base using production rules. Other techniques for knowledge base representation include logical representation, semantic network representation, and frame representation. We chose production rules and in particular ripple-down rules to represent the knowledge base. Following this technique, we built an RDR tree, as shown in Figure 2, where each node contains a logical condition, explanation, and conclusion. A node can have at most two children.

### 3.2. Verification of Knowledge Base

Two main categories of knowledge were verified. First, the daily allowable limits of the three BCAAs, protein, energy, and fluid were evaluated according to age (0–6 months), as listed in Table 1.

Second, the nutrient content of six types of formulas was collected, as listed in Table 2. This table shows commercial (first three rows) and medical (last three rows) formulas suitable for children with IEMs. The medical formulas do not contain BCAAs, indicating their suitability for MSUD patients. However, medical formulas are generally more expensive than commercial or dietary ones owing to their sustainability and production under supervision of dietitians, who know the correct contents for required metabolic rates and specifications, being more useful when considering the patient’s body, characteristics, and BCAA requirements.

Regarding the recommended intake of BCAAs, protein, energy, and fluid according to age (0–6 months) shown in Table 2, two metabolic dietitians expressed the need for a standard unit, as it could help MSUD patients’ caregivers to easily control the intake of the formulas listed in Table 2. The results of standardization are reported as equations in Appendix A. This appendix explains the dietary formulations where the dosage amount is calculated according to the selected commercial formula’s and infant’s weight (w). Additionally, the rules for diet plans are detailed in Appendix B. This appendix outlines the feeding rules based on the patient’s daily feeding plan after standardizing the formula scoop size. The doses per type of formula are specified in units of 5 g, which is equivalent to one spoonful (provided by the metabolic dietitian). This dose unit is used to facilitate complex calculations of the BCAAs and dosage. The resultant outlined tables recommend that, based on a 5 g scoop size, the nutrient content outlined in Table 2 and the rules specified in Appendix A and Appendix B can be considered to build the knowledge base of MSUD Baby Buddy.

### 3.3. Validation of Knowledge-Based Tool

We validated the tool usability using SUS for the front-end part and unit and integration testing for the back-end part of the tool. For the front-end part, we tested the interfaces on 10 MSUD caregivers *(n* = 10). These caregivers completed the SUS survey and provided feedback through open-ended questions. All respondents were parents (eight women and two men) of MSUD patients. Based on the answers to the usability questionnaire, the average SUS score was 84.25, indicating that MSUD Baby Buddy is usable and may help caregivers of MSUD patients to determine the appropriate doses of formula based on four variables: age, weight, plasma BCAAs, and formula preference.

**Figure 3:**
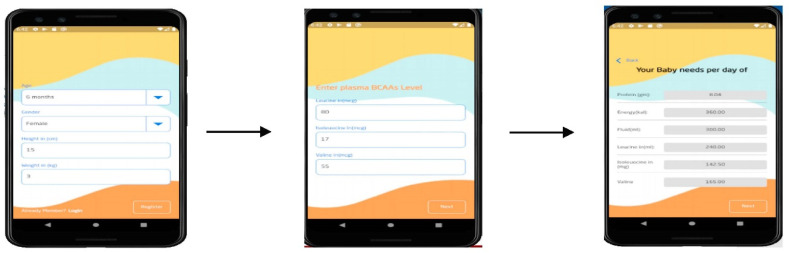
MSUD patient profile.

**Figure 4:**
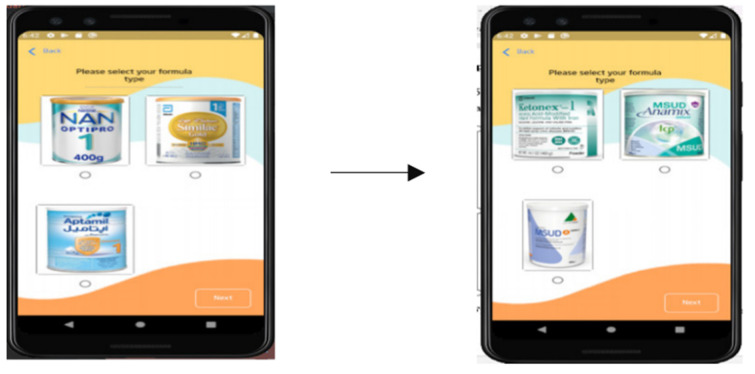
Allowing the caregivers to choose their preferred medical/commercial formulas.

**Figure 5:**
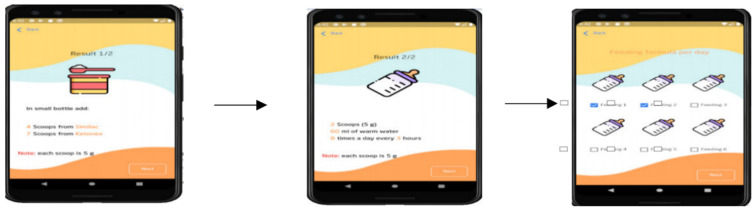
Recommending the appropriate daily doses of formula.

The validation of the back-end part of the tool was carried out using unit testing for the three main functions of the knowledge-based system. The first function enables MSUD patients’ caregivers to provide required data and fill out questionnaires and forms related to the input variables, as shown in Figure 3. The second function allows users to select their preferred formula from medical and commercial options (Figure 4). The validity is checked through studying and researching the formula composition for suitability with the patient’s body and reaction on consumption according to the recommended daily intake. Finally, based on calculations, the recommended daily intake of energy, protein, and allowable limits of BCAAs (i.e., valine, leucine, and isoleucine) is shown on the interface (Figure 5). The information about unit testing for the three functions is listed in Table 3.

These results, together, suggest that MSUD Baby Buddy is technically valid and can deliver the three main functionalities outlined in Figure 3, Figure 4, and Figure 5, respectively. Brief interviews with caregivers and their responses to the open-ended questions revealed that continuous monitoring of the metabolic rate is considered essential for preventing severe side effects from overtaking a dangerous BCAA. In addition, caregivers considered that pediatricians are very busy, increasing the waiting time of patients at the hospital, whereas quality time is reduced for families with less access to specialists whenever they require appointments in between. Moreover, caregivers considered the waiting times as tiring, and that patients need to manage the amino acid calculations at home because there are no adequate facilities available at hospitals with comfortable environments for the pediatric patients.

Using assistive tools such as the proposed MSUD Baby Buddy may increase confidence and reduce anxiety and fear of caregivers in managing the disease. In addition, practitioners may schedule appointments and follow-ups in longer intervals. Hence, despite limited access to specialists, continuous monitoring may be enabled by using tools such as MSUD Baby Buddy. Moreover, metabolic and diet control for BCAAs may be easily achieved using a diet-tracking app if patients or parents are educated about the formulas. The role of dietitians has been concerned with follow-ups, and interviews with parents in this study showed that the importance of a metabolic dietitian has been overemphasized by health agents. Nevertheless, regular appointments with a dietitian for monitoring the blood levels of amino acids in children are common. In fact, close monitoring and regular appointments with practitioners are necessary for children to suitably control MSUD. We also found service and compliance problems that should be investigated by families regarding nutritional monitoring.

## 4. Discussion

MSUD is an IEM that exposes infant patients to a great risk of suffering from hypercatabolism [3,4]. Moreover, MSUD can cause metabolic crises and learning difficulties. If left untreated, MSUD patients may deteriorate, slip into coma, and die, in the worst case. Managing the diet of an MSUD patient primarily affects caregivers, who are responsible for maintaining a low-protein diet. After a newborn is discharged from hospital, caregivers suffer from a psychological burden caused by the MSUD diagnosis and responsibility for the dietary therapy [4]. Dietitians who treat MSUD should support the families psychologically, considering the four main aspects of IEMs as rare, metabolic, chronic, and genetic diseases [4]. The child’s health condition depends on the care provided by the family at home, and self-tracking mobile health solutions can greatly help in treatment [13]. From a technical perspective, self-tracking involves digital systems and devices that allow users to collect, analyze, and reflect on data [14,15]. In this sense, self-tracking may help caregivers to pay special attention to the daily needs of MSUD patients. MSUD Baby Buddy allows users to regularly update the patient’s weight, age, and plasma BCAA levels to properly adjust the recommended daily intake of protein, BCAAs, and formula doses.

The customization of dietary guidance has been widely studied to help to manage diet-related chronic diseases or maintain a healthy lifestyle [16]. In particular, personalized nutrition provides dietary recommendations according to specific biological requirements based on a person’s health status and goals [17]. Personalized nutrition is delineated by three elements: (1) science and data, (2) professional education and training, and (3) guidance and therapeutics. Similarly, we have developed a technological solution that further increases personalized nutrition guidance, products, and services by focusing on MSUD patients. MSUD is an IEM for which protein and BCAA intake must be carefully controlled. Solutions based on artificial intelligence and decision making will likely help caregivers to closely monitor the patient’s protein intake based on medical and commercial formulas.

Additional research is required to expand the knowledge base and include other types of commercial and medical formulas. The six types of formulas included in this research were selected based on a preliminary exploratory questionnaire intended to identify the common types used in a city. However, these formulas are not standard. For the knowledge base to be applicable to a larger population of MSUD patients, we must consider other standard types of formulas. In addition, breast milk must be included in the knowledge base. Some mothers indicated that they prefer to breastfeed their children but are concerned with the BCAA contents, which vary from one mother to another. Although an average content can be obtained, careful monitoring by metabolic dietitians and clinical nutritionists will be required for safe breastfeeding.

## 5. Conclusions

The MSUD Baby Buddy app aims to provide caregivers accurate recommended daily intake of proteins, BCAAs, and fluids for infants 0–6 months of age through calculations based on the MSUD patient’s age, weight, and plasma BCAA levels. The knowledge-based system allows caregivers to feed patients with their preferred medical or commercial formulas and provide adequate intake of protein, energy, and fluids. The average system usability score is 84.25, indicating that MSUD Baby Buddy is technically usable and would help caregivers to easily determine the recommended formula dosage based on the MSUD patient’s biometric data and preferred formulas. This tool seems promising to support caregivers of MSUD patients in independently tracking protein intake and reducing the required number of visits to pediatricians and metabolic dietitians.

## Figures and Tables

**Table 1:** Daily recommended intake of BCAAs, protein, and fluid for MSUD patients.

Age (months)	Isoleucine (mg)	Leucine (mg)	Valine (mg)	Protein (gm)	Energy (kcal)	Fluid (mL)
1	40	66.5	46.5	3.45	120	100
2	48	79.5	55.5	3.34	120	100
3	56	92.5	64.5	3.16	120	100
4	48.5	56	56	3	120	100
5	40.5	68	47	2.84	120	100
6	47.5	80	55	2.68	120	100

**Table 2:** Nutrient content of six common formulas.

Formula	Leucine (mg)	Isoleucine (mg)	Valine (mg)	Protein (gm)	Energy (kcal)
Nan 1^®^	106.4	51.6	53.6	12.3	506
Similac^®^	1079	573	641	10.83	526
Aptamil^®^	1020	480	520	4.7	485
Ketonex-1^®^	0	0	0	15	480
Anamix^®^	0	0	0	13.1	466
Comida^®^	0	0	0	12	205

**Table 3:** Unit testing for three functions of MSUD Baby Buddy.

Unit Test ID	Description	Input	Expected Result	Actual Result	Pass/Fail
1	Verify input plasma BCAA by inserting valid data	Leucine: 80 Isoleucine: 17 Valine: 55	After caregiver submits plasma BCAA, the system shows recommended daily intake	Display recommended daily intake for patient	Pass
2	Verify input plasma BCAA by inserting invalid data	Leucine: 80 Isoleucine: 17 Valine: –	Display error message in invalid field	Display error message in all fields	Pass
3	Verify correct recommended daily intake based on weight and age of patient	No input data	Display necessary limits for BCAAs, protein, energy, and fluid for patient	Display necessary limits for BCAAs, protein, energy, and fluid for patient	Pass
4	Select commercial formula	Similac^®^ (commercial formula) selected from image	Caregivers select preferred formula	Show recommended daily intake of formula	Pass
5	Select medical formula	Ketonex-1 (medical formula) selected from image	Caregivers select preferred formula	Show recommended daily intake of formula	Pass
6	Select commercial formula	No selection	Display error message “Please select formula “	Display error message “Please select formula”	Pass
7	Select medical formula	No selection	Display error message “Please select formula”	Display error message “Please select formula”	Pass

## Data Availability

The following link is provided to the project repository. It is public for everyone to install the software and start using it: https://github.com/malraegi/MSUD_Baby_Buddy.git, accessed on 2 September 2022.

## Abbreviations

MSUD	Maple syrup urine disease
IEM	Inborn error of metabolism
RDRs	Ripple-down rules
SUS	System usability score

## Appendix A

This appendix explains the dietary formulations where the dosage amount is calculated according to the selected commercial formula’s and infant’s weight (w).

Similac

$$\mathrm{VAL}\_\mathrm{sim}\_\mathrm{res}=\frac{100\times \left(\mathrm{val}\;\mathrm{with}\;w\right)}{641}$$

$$\mathrm{LEU}\_\mathrm{sim}\_\mathrm{res}=\frac{100\times \left(\mathrm{leu}\;\mathrm{with}\;w\right)}{1079}$$

$$\mathrm{Protein}\_\mathrm{sim}\_\mathrm{res}=\frac{10.83\times \mathrm{VAL}\_\mathrm{sim}\_\mathrm{res}\;}{100}$$

Nan1

$$\mathrm{VAL}\_\mathrm{Nan}\_\mathrm{res}=\frac{100\times \left(\mathrm{val}\;\mathrm{with}\;w\right)}{53.6}$$

$$\mathrm{LEU}\_\mathrm{Nan}\_\mathrm{res}=\frac{100\times \left(\mathrm{leu}\;\mathrm{with}\;w\right)}{106.4}$$

$$\mathrm{Protein}\_\mathrm{Apt}\_\mathrm{res}=\frac{12.3\times \mathrm{VAL}\_\mathrm{Nan}\_\mathrm{res}\;}{100}$$

Aptamil

$$\mathrm{VAL}\_\mathrm{Apt}\_\mathrm{res}=\frac{100\times \left(\mathrm{val}\;\mathrm{with}\;w\right)}{520}$$

$$\mathrm{LEU}\_\mathrm{Apt}\_\mathrm{res}=\frac{100\times \left(\mathrm{leu}\;\mathrm{with}\;w\right)}{1020}$$

$$\mathrm{Protein}\_\mathrm{Apt}\_\mathrm{res}=\frac{4.7\times \mathrm{VAL}\_\mathrm{Apt}\_\mathrm{res}\;}{100}$$

Dosage calculation according to selected medical formula:

Ketonix-1

Pro_keto = protein content of user-selected commercial formula − plasma protein content and weight of patient

$$\mathrm{Protein}\_\mathrm{keto}\_\mathrm{res}=\frac{100\times \mathrm{Pro}\_\mathrm{keto}}{15}$$

$$\mathrm{Energy}\_\mathrm{keto}\_\mathrm{res}=\frac{480\times \mathrm{Protein}\_\mathrm{keto}\_\mathrm{res}}{100}$$

Anamix

Pro_Ana = protein content of user-selected commercial formula − plasma protein content and weight of patient

$$\mathrm{Protein}\_\mathrm{Ana}\_\mathrm{res}=\frac{100\times \mathrm{Ana}\_\mathrm{keto}}{13.1}$$

$$\mathrm{Energy}\_\mathrm{Ana}\_\mathrm{res}=\frac{466\times \mathrm{Protein}\_\mathrm{Ana}\_\mathrm{res}}{100}$$

Comida

Pro_Com = protein content of user-selected commercial formula − plasma protein content and weight of patient

$$\mathrm{Protein}\_\mathrm{Com}\_\mathrm{res}=\frac{100\times \mathrm{Com}\_\mathrm{keto}}{12}$$

$$\mathrm{energy}\_\mathrm{Com}\_\mathrm{res}=\frac{205\times \mathrm{protein}\_\mathrm{Com}\_\mathrm{res}}{100}$$

## Appendix B

This appendix outlines the feeding rules based on the patient’s daily feeding plan after standardizing the formula scoop size.

Fluid_b= 100 × w

Feeding = Fluid_b/5

If (Feeding == 25) {

     ¾ scoops (5 g)

     25 mL of warm water

     8 times a day every 3 h}

If (Feeding == 30) {

     1 scoop (5 g)

     30 mL of warm water

     8 times a day every 3 h}

If (Feeding == 40) {

     1 ¾ scoops (5 g)

     40 mL of warm water

     8 times a day every 3 h}

If (Feeding == 60) {

     2 scoops (5 g)

     60 mL of warm water

     8 times a day every 3 h}

If (Feeding == 100) {

     3 scoops (5 g)

     100 mL of warm water

     8 times a day every 3 h}

If (Feeding == 120) {

     4 scoops (5 g)

     120 mL of warm water

     8 times a day every 3 h}

If (Feeding == 160) {

     5 scoops (5 g)

     160 mL of warm water

     8 times a day every 3 h}

If (Feeding == 180) {

     6 scoops (5 g)

     180 mL of warm water

8 times a day every 3 h}

## References

[1] Bharadwaj A., Wahi N., Saxena A. (2021). Occurrence of Inborn Errors of Metabolism in Newborns, Diagnosis and Prophylaxis. Endocr. Metab. Immune Disord. Drug Targets.

[2] Strauss K.A., Puffenberger E.G., Carson V.J., Adam M.P., Everman D.B., Mirzaa G.M., Pagon R.A., Wallace S.E., Bean L.J., Gripp K.W., Amemiya A. (1993). Maple Syrup Urine Disease. GeneReviews^®^.

[3] Boyer S.W., Barclay L.J., Burrage L.C. (2015). Inherited Metabolic Disorders. Nutr. Clin. Pract..

[4] Difficulties in Daily Life and Associated Factors, and QoL of Children with Inherited Metabolic Disease and Their Parents in Japan: A Literature Review|SpringerLink. https://link-springer-com.ccl.idm.oclc.org/chapter/10.1007/8904_2016_573.

[5] Jimenez G., Lum E., Car J. (2019). Examining Diabetes Management Apps Recommended From a Google Search: Content Analysis. JMIR MHealth UHealth.

[6] Ho G., Ueda K., Houben R.F., Joa J., Giezen A., Cheng B., van Karnebeek C.D. (2016). Metabolic Diet App Suite for inborn errors of amino acid metabolism. Mol. Genet. Metab..

[7] DietAssistant for PKU. https://www.dietassistant.net/en/.

[8] Acosta P.B., Yannicelli S. (2001). Abbott Laboratories, and Ross Products Division. Nutrition Support Protocols: The Ross Metabolic Formula System.

[9] Induction of Ripple-Down Rules Applied to Modeling Large Databases|SpringerLink. https://link.springer.com/article/10.1007/BF00962234.

[10] Usability Measurement of Mobile Applications with System Usability Scale (SUS)|SpringerLink. https://link-springer-com.ccl.idm.oclc.org/chapter/10.1007/978-3-030-03317-0_32.

[11] AlGhannam B.A., Albustan S.A., Al-Hassan A.A., Albustan L.A. (2018). Towards a Standard Arabic System Usability Scale: Psychometric Evaluation using Communication Disorder App. Int. J. Human–Computer Interact..

[12] Ellims M., Bridges J., Ince D.C. (2006). The Economics of Unit Testing. Empir. Softw. Eng..

[13] Psychosocial Issues in Families Affected by Maple Syrup Urine Disease—PubMed. https://pubmed.ncbi.nlm.nih.gov/17703353/.

[14] Full Article: Self-Tracking, Health and Medicine. https://www.tandfonline.com/doi/full/10.1080/14461242.2016.1228149.

[15] French M., Smith G. (2013). ‘Health’ Surveillance: New Modes of Monitoring Bodies, Populations, and Polities. Critical Public Health.

[16] Lomborg S., Frandsen K. (2016). Self-tracking as communication. Inf. Commun. Soc..

[17] Trends in Personalized Nutrition—Google Books. https://books.google.com.sa/books?hl=en&lr=&id=hfGZDwAAQBAJ&oi=fnd&pg=PP1&dq=Trends+in+personalized+nutrition&ots=A3aMtQv-ZL&sig=Ljcfc21CbUZch8ACtO8flpMYTc0&redir_esc=y#v=onepage&q=Trends%20in%20personalized%20nutrition&f=false.

